# Supplementary material for: Habitual Mask Wearing as Part of COVID-19 Control in Japan: An Assessment Using the Self-Report Habit Index
Source: Behav Sci (Basel). 2023 Nov 19;13(11):951. doi: 10.3390/bs13110951 (PMC10669277; doi:10.3390/bs13110951)
Supplement: Supplementary file 1 [file behavsci-13-00951-s001.zip › MaskSurvey_Questionnaire_Li_17Nov2023.pdf]

Supplemental File S1: Questionnaire – English Version

Survey on Awareness and Automatism

of Infection Prevention Measures

Dear participant,

This survey will examine your current state of mask wearing to prevent COVID-19 infection in indoor environments and on public transport, and will contribute to future measures to prevent its spread.

All responses will be handled in a privacy-protected manner. The results of this survey may be reused for research purposes, while ensuring anonymity.

This survey examines the current use of masks in indoor environments.

Even if you are unsure, please select responses that are closest to your intended meaning.

Please fill in the following answer fields.

[Definition]

In this survey, we will inquire about masks in general, including all types.

However, “non-woven fabric masks” will appear in some questions. Non-woven fabric masks are defined as follows:

・ Masks sold in retail stores (such as supermarkets, pharmacies, convenience stores, etc.) for household, medical, industrial use, etc., consisting of “non-woven fabric” that is woven without using threads.

・ 3D masks (non-woven fabric) as shown in the illustration below are also considered non-woven fabric masks.

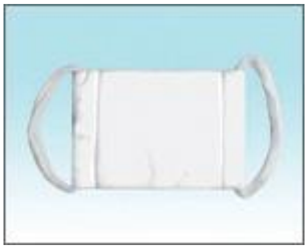

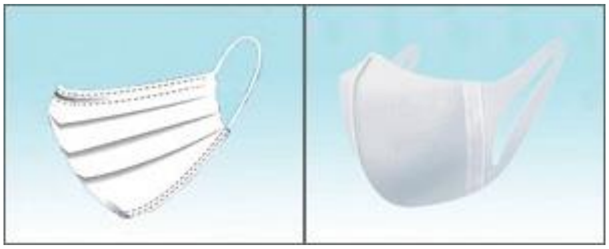

Gauze masks Non-woven fabric masks (flat and 3D masks)

(These images are from the following website: https://www.jhpia.or.jp/product/mask/index.html#q4)

・ Non-woven fabric masks include not only disposable masks but also high performance masks that can be washed and reused.

Please provide information regarding the use of masks (any type of mask is acceptable) in the indoor space where you spend the most time during the day, such as your workplace, school, nursing home, etc.

This survey does not include your home. If your home is where you spend the most time, please answer regarding the use of masks outside your home where you spend the most time with people other than family members.

Q1. At present, do you wear a mask in the indoor space (other than your home) where you spend the most time during the day?

□ Usually (go to Question 2)

□ Sometimes (go to Question 2)

□ Not at all (go to Question 3)

Q2. This question is for those who wear masks in indoor spaces (other than their home) where they spend the most time during the day. At present, which type of mask do you use the most?

□ Non-woven fabric masks (including surgical masks sold at convenience stores)

□ Gauze masks (masks made mainly of layered cotton fabric)

□ Urethane masks (relatively breathable masks that can be purchased at clothing stores, etc.)

□ Other (including self-made fabric masks)

Q3. In which indoor space, besides your home, do you currently spend the most time during the day?

□ Office (excluding the options below)

□ Kindergarten, nurseries, or other educational institutions, such as schools and cram schools.

□ Medical facilities and care facilities, such as hospitals and daytime care services for older adults.

□ Stores and retail shops, such as supermarkets, shopping malls, and produce stands.

□ Public facilities, such as city halls and civic centers.

□ Other (please specify the indoor space other than your home) _________

Q4. Please rate the following 6 items on a 5-point scale from "1 (not at all)" to "5 (very much)" regarding the reasons why you wear a mask at present.

Q4-1. Do you think your symptoms would be serious if you were infected with COVID-19?

□ 1. not at all □ 2 □ 3 □ 4 □ 5. very much

Q4-2. Do you think wearing a mask will prevent you from being infected with COVID-19?

□ 1. not at all □ 2 □ 3 □ 4 □ 5. very much

Q4-3. Do you believe that wearing a mask will prevent an infected person from transmitting COVID-19 to others?

□ 1. not at all □ 2 □ 3 □ 4 □ 5. very much

Q4-4. Do you agree with the statement "Anything is fine, let's take whatever measures we can take against COVID-19"?

□ 1. not at all □ 2 □ 3 □ 4 □ 5. very much

Q4-5. When you see people wearing masks, do you feel that you should wear one too?

□ 1. not at all □ 2 □ 3 □ 4 □ 5. very much

Q4-6. Do you feel wearing a mask can reduce anxiety?

□ 1. not at all □ 2 □ 3 □ 4 □ 5. very much

In the remaining part of the survey, we will ask you about your mask-wearing habits.

First, we will ask about wearing masks indoors. By “indoors,” we are referring to the places in which you spend the most time during the day, excluding your home.

Please answer the following question about indoor spaces, excluding areas where you spend time while using public transport (such as trains and buses). We will ask about mask wearing while using public transport later in the questionnaire.

Q5 Currently, regarding mask-wearing in indoor spaces, please rate the following 12 items on a 7-point scale from “1 (disagree)” to “7 (agree)”.

Q5-1. I wear masks in indoor places (other than at home) frequently.

□ 1. disagree □ 2 □ 3 □ 4 □ 5 □ 6 □ 7. agree

Q5-2. I wear masks in indoor places (other than at home) automatically.

□ 1. disagree □ 2 □ 3 □ 4 □ 5 □ 6 □ 7. agree

Q5-3. I wear masks in indoor places (other than at home) without having to consciously remember.

□ 1. disagree □ 2 □ 3 □ 4 □ 5 □ 6 □ 7. agree

Q5-4. If I don’t wear masks in indoor places (other than at home), I feel weird.

□ 1. disagree □ 2 □ 3 □ 4 □ 5 □ 6 □ 7. agree

Q5-5. I wear masks in indoor places (other than at home) without thinking.

□ 1. disagree □ 2 □ 3 □ 4 □ 5 □ 6 □ 7. agree

Q5-6. It would require effort to not wear masks in indoor places (other than at home).

□ 1. disagree □ 2 □ 3 □ 4 □ 5 □ 6 □ 7. agree

Q5-7. Wearing masks in indoor places (other than at home) is part of my daily routine.

□ 1. disagree □ 2 □ 3 □ 4 □ 5 □ 6 □ 7. agree

Q5-8. I start wearing masks in indoor places (other than at home) before I realize I am doing it.

□ 1. disagree □ 2 □ 3 □ 4 □ 5 □ 6 □ 7. agree

Q5-9. I would find it hard not to wear masks in indoor places (other than at home).

□ 1. disagree □ 2 □ 3 □ 4 □ 5 □ 6 □ 7. agree

Q5-10. I have no need to think about wearing masks in indoor places (other than at home).

□ 1. disagree □ 2 □ 3 □ 4 □ 5 □ 6 □ 7. agree

Q5-11. Wearing masks in indoor places (other than at home) is typically "me".

□ 1. disagree □ 2 □ 3 □ 4 □ 5 □ 6 □ 7. agree

Q5-12. I have been wearing masks in indoor places (other than at home) for a long time.

□ 1. disagree　□ 2　□ 3　□ 4　□ 5　□ 6　□ 7. agree

Next, we will ask about your mask-wearing habits when using public transport.

Here, public transport refers to transportation facilities such as trains and buses that can be freely used by anyone who pays the specified fare.

Q6. Which type of public transportation do you currently use most frequently?

□ Train (including monorails, trams, and steam trains)

□ Bus (including community buses)

□ Aircraft

□ Boat

□ Others (please specify the public transportation than the above options) ___________

Below, we would like to ask about wearing masks when using public transport.

Here, "public transport" refers to transportation systems such as trains and buses that can be freely used by paying a predetermined fare by an unspecified number of people.

Q7 Regarding mask-wearing on public transport, please rate the following 12 items on a 7-point scale from "1 (disagree)" to "7 (agree)".

Q7-1. I wear masks on public transportation frequently.

□ 1. disagree □ 2 □ 3 □ 4 □ 5 □ 6 □ 7. agree

Q7-2. I wear masks on public transportation automatically.

□ 1. disagree □ 2 □ 3 □ 4 □ 5 □ 6 □ 7. agree

Q7-3. I wear masks on public transportation without having to consciously remember.

□ 1. disagree □ 2 □ 3 □ 4 □ 5 □ 6 □ 7. agree

Q7-4. If I don't wear masks on public transportation, I feel weird.

□ 1. disagree □ 2 □ 3 □ 4 □ 5 □ 6 □ 7. agree

Q7-5. I wear masks on public transportation without thinking.

□ 1. disagree □ 2 □ 3 □ 4 □ 5 □ 6 □ 7. agree

Q7-6. It would require effort to not wear masks on public transportation.

□ 1. disagree □ 2 □ 3 □ 4 □ 5 □ 6 □ 7. agree

Q7-7. Wearing masks on public transportation is part of my daily routine.

□ 1. disagree □ 2 □ 3 □ 4 □ 5 □ 6 □ 7. agree

Q7-8. I start wearing masks on public transportation before I realize I am doing it.

□ 1. disagree □ 2 □ 3 □ 4 □ 5 □ 6 □ 7. agree

Q7-9. I would find it hard not to wear masks on public transportation.

□ 1. disagree □ 2 □ 3 □ 4 □ 5 □ 6 □ 7. agree

Q7-10. I have no need to think about wearing masks on public transportation.

□ 1. disagree □ 2 □ 3 □ 4 □ 5 □ 6 □ 7. agree

Q7-11. Wearing masks on public transportation is typically "me".

□ 1. disagree □ 2 □ 3 □ 4 □ 5 □ 6 □ 7. agree

Q7-12. I have been wearing masks on public transportation for a long time.

□ 1. disagree □ 2 □ 3 □ 4 □ 5 □ 6 □ 7. agree
