# Supplementary material for: Habitual Mask Wearing as Part of COVID-19 Control in Japan: An Assessment Using the Self-Report Habit Index
Source: Behav Sci (Basel). 2023 Nov 19;13(11):951. doi: 10.3390/bs13110951 (PMC10669277; doi:10.3390/bs13110951)
Supplement: Supplementary file 1 [file behavsci-13-00951-s001.zip › MaskSurvey_SupplementaryMaterial_Li_06Oct2023.pdf]

Supplementary Material

Table S1: Correlations among variables regarding mask usage

	Severity	Protection	Prevention	Impulsion	Norm	Relief	Frequency
Severity	-						
Protection	0.31	-					
Prevention	0.27	0.64	-				
Impulsion	0.39	0.49	0.40	-			
Norm	0.33	0.44	0.36	0.63	-		
Relief	0.33	0.58	0.48	0.57	0.63	-	
Frequency	0.14	0.19	0.16	0.24	0.30	0.23	-
M	3.17	3.38	3.43	3.76	3.99	3.65	2.79
SD	1.11	1.01	1.03	1.04	1.05	1.07	0.52
N = 2640							

M: Means; *SD*: Standard Deviation; *N*: number of samples

Table S2: Association between mask wearing and psychological reasons in female participants

Variable	Standardized coefficient	*p*-value
Severity	0.03 (0.02)	0.30
Protection	0.05 (0.03)	0.17
Prevention	-0.02 (0.03)	0.55
Impulsion	0.03 (0.03)	0.43
Norm	0.14 (0.03)	<0.0001
Relief	0.06 (0.03)	0.06

Frequency of mask usage as explained by six variables. Dependent variable: the frequency of mask wearing; independent variable: six psychological reasons variables. Standard errors are shown in brackets. R^2 = 0.06, R^2(adj) = 0.06, root mean square error (RSME) = 0.44. The test level is α = 0.05; *p*-value is calculated by multiple linear regression.

Table S3: Association between mask wearing and psychological reasons in male participants

Variable	Standardized coefficient	*p*-value
Severity	0.01 (0.03)	0.82
Protection	0.03 (0.04)	0.42
Prevention	0.06 (0.04)	0.11
Impulsion	0.06 (0.04)	0.12
Norm	0.27 (0.04)	<0.0001
Relief	-0.04 (0.04)	0.38

Frequency of mask usage as explained by six variables. Dependent variable: the frequency of mask wearing; independent variable: six psychological reasons variables. Standard errors are shown in brackets. $R^2 = 0.11$, $R^2(adj) = 0.11$, root mean square error (RSME) = 0.53. The test level is $\alpha = 0.05$; *p*-value is calculated by multiple linear regression.

Table S4: Association between mask wearing and psychological reasons in married participants

Variable	Standardized coefficient	*p*-value
Severity	0.01 (0.02)	0.78
Protection	0.05 (0.04)	0.17
Prevention	-0.01 (0.03)	0.71
Impulsion	0.04 (0.03)	0.25
Norm	0.20 (0.04)	<0.0001
Relief	0.02 (0.04)	0.61

Frequency of mask usage as explained by six variables. Dependent variable: the frequency of mask wearing; independent variable: six psychological reasons variables. Standard errors are shown in brackets. R^2 = 0.07, R^2(adj) = 0.07, root mean square error (RSME) = 0.47. The test level is $\alpha = 0.05$; *p*-value is calculated by multiple linear regression.

Table S5: Association between mask wearing and psychological reasons in unmarried participants

Variable	Standardized coefficient	*p*-value
Severity	0.03 (0.03)	0.39
Protection	0.03 (0.04)	0.49
Prevention	0.06 (0.04)	0.13
Impulsion	0.06 (0.04)	0.10
Norm	0.24 (0.04)	<0.0001
Relief	0.01 (0.04)	0.88

Frequency of mask usage as explained by six variables. Dependent variable: the frequency of mask wearing; independent variable: six psychological reasons variables. Standard errors are shown in brackets. $R^2 = 0.12$, $R^2(adj) = 0.12$, root mean square error (RSME) = 0.51. The test level is $\alpha = 0.05$; *p*-value is calculated by multiple linear regression.

Table S6: Association between mask wearing and psychological reasons in participants who have at least one child

Variable	Standardized coefficient	*p*-value
Severity	0.01 (0.03)	0.83
Protection	0.03 (0.04)	0.46
Prevention	-0.03 (0.03)	0.35
Impulsion	0.06 (0.04)	0.14
Norm	0.21 (0.04)	<0.0001
Relief	0.02 (0.04)	0.60

Frequency of mask usage as explained by six variables. Dependent variable: the frequency of mask wearing; independent variable: six psychological reasons variables. Standard errors are shown in brackets. $R^2 = 0.07$, $R^2(adj) = 0.07$, root mean square error (RSME) = 0.48. The test level is $\alpha = 0.05$; *p*-value is calculated by multiple linear regression.

Table S7: Association between mask wearing and psychological reasons in participants who do not have children

Variable	Standardized coefficient	*p*-value
Severity	0.03 (0.03)	0.29
Protection	0.05 (0.04)	0.20
Prevention	0.06 (0.03)	0.06
Impulsion	0.05 (0.04)	0.17
Norm	0.23 (0.03)	<0.0001
Relief	0.01 (0.04)	0.80

Frequency of mask usage as explained by six variables. Dependent variable: the frequency of mask wearing; independent variable: six psychological reasons variables. Standard errors are shown in brackets. $R^2 = 0.12$, $R^2(adj) = 0.12$, root mean square error (RSME) = 0.50. The test level is $\alpha = 0.05$; *p*-value is calculated by multiple linear regression.

Table S8: Association between mask wearing and psychological reasons in participants aged 20–29 years old

Variable	Standardized coefficient	*p*-value
Severity	-0.01 (0.05)	0.83
Protection	0.02 (0.07)	0.74
Prevention	0.12 (0.06)	0.05
Impulsion	0.00 (0.07)	0.97
Norm	0.28 (0.06)	<0.0001
Relief	-0.04 (0.06)	0.45

Frequency of mask usage as explained by six variables. Dependent variable: the frequency of mask wearing; independent variable: six psychological reasons variables. Standard errors are shown in brackets. R^2 = 0.12, R^2(adj) = 0.10, root mean square error (RSME) = 0.52. The test level is $\alpha = 0.05$; *p*-value is calculated by multiple linear regression.

Table S9: Association between mask wearing and psychological reasons in participants aged 30–39 years old

Variable	Standardized coefficient	*p*-value
Severity	-0.01 (0.04)	0.87
Protection	0.01 (0.06)	0.83
Prevention	-0.02 (0.05)	0.69
Impulsion	0.10 (0.06)	0.06
Norm	0.23 (0.06)	<0.0001
Relief	-0.01 (0.06)	0.91

Frequency of mask usage as explained by six variables. Dependent variable: the frequency of mask wearing; independent variable: six psychological reasons variables. Standard errors are shown in brackets. $R^2 = 0.11$, $R^2(\text{adj}) = 0.10$, root mean square error (RSME) = 0.47. The test level is $\alpha = 0.05$; *p*-value is calculated by multiple linear regression.

Table S10: Association between mask wearing and psychological reasons in participants aged 40–49 years old

Variable	Standardized coefficient	*p*-value
Severity	0.08 (0.05)	0.12
Protection	0.10 (0.07)	0.14
Prevention	0.04 (0.07)	0.58
Impulsion	0.12 (0.06)	0.07
Norm	0.20 (0.06)	<0.01
Relief	-0.03 (0.07)	0.65

Frequency of mask usage as explained by six variables. Dependent variable: the frequency of mask wearing; independent variable: six psychological reasons variables. Standard errors are shown in brackets. R^2 = 0.14, R^2(adj) = 0.12, root mean square error (RSME) = 0.52. The test level is $\alpha = 0.05$; *p*-value is calculated by multiple linear regression.

Table S11: Association between mask wearing and psychological reasons in participants aged 50–59 years old

Variable	Standardized coefficient	*p*-value
Severity	0.10 (0.05)	0.07
Protection	-0.03 (0.07)	0.70
Prevention	-0.01 (0.06)	0.85
Impulsion	0.01 (0.07)	0.85
Norm	0.17 (0.07)	0.01
Relief	0.16 (0.07)	0.03

Frequency of mask usage as explained by six variables. Dependent variable: the frequency of mask wearing; independent variable: six psychological reasons variables. Standard errors are shown in brackets. $R^2 = 0.11$, $R^2(\text{adj}) = 0.10$, root mean square error (RSME) = 0.48. The test level is $\alpha = 0.05$; *p*-value is calculated by multiple linear regression.

Table S12: Association between mask wearing and psychological reasons in participants aged 60–69 years old

Variable	Standardized coefficient	*p*-value
Severity	0.04 (0.04)	0.34
Protection	0.10 (0.07)	0.15
Prevention	0.00 (0.06)	0.96
Impulsion	0.11 (0.06)	0.08
Norm	0.13 (0.07)	0.07
Relief	-0.03 (0.07)	0.70

Frequency of mask usage as explained by six variables. Dependent variable: the frequency of mask wearing; independent variable: six psychological reasons variables. Standard errors are shown in brackets. R^2 = 0.08, R^2(adj) = 0.07, root mean square error (RSME) = 0.46. The test level is $\alpha = 0.05$; *p*-value is calculated by multiple linear regression.

Table S13: Association between mask wearing and psychological reasons in participants aged ≥ 70 years old

Variable	Standardized coefficient	*p*-value
Severity	-0.02 (0.05)	0.62
Protection	0.05 (0.07)	0.50
Prevention	-0.04 (0.06)	0.50
Impulsion	-0.03 (0.07)	0.67
Norm	0.31 (0.07)	<0.0001
Relief	0.09 (0.06)	0.16

Frequency of mask usage as explained by six variables. Dependent variable: the frequency of mask wearing; independent variable: six psychological reasons variables. Standard errors are shown in brackets. $R^2 = 0.10$, $R^2(adj) = 0.08$, root mean square error (RSME) = 0.48. The test level is $\alpha = 0.05$; *p*-value is calculated by multiple linear regression.

Table S14: Self-report habit index scores stratified in different groups

SRHI score in indoor space		Estimate†	SRHI score on public transport		Estimate†
Sex			Sex		
	Male	61.5 (±16.5)		Male	63.6 (±19.8)
	Female	68.2 (±14.2)		Female	70.8 (±16.1)
Marital status			Marital status		
	Married	66.8 (±14.8)		Married	69.1 (±17.8)
	Unmarried	62.5 (±16.5)		Unmarried	64.9 (±18.8)
Have at least one child			Have at least one child		
	Yes	67.3 (±14.9)		Yes	69.1 (±18.2)
	No	62.7 (±16.2)		No	65.5 (±18.4)
Age group (years)			Age group (years)		
	20–29	61.9 (±15.3)		20–29	65.2 (±16.8)
	30–39	61.4 (±16.2)		30–39	62.7 (±19.8)
	40–49	62.0 (±16.8)		40–49	65.7 (±17.6)
	50–59	64.9 (±15.2)		50–59	67.8 (±17.6)
	60–69	68.2 (±14.6)		60–69	69.6 (±18.6)
	≥70	70.7 (±13.7)		≥70	72.1 (±18.4)
Indoor space that most frequently visited			Public transport most frequently used		
	Office	62.9 (±15.8)		Train	69.5 (±15.3)
	School	66.2 (±14.8)		Bus	70.5 (±15.3)
	Healthcare Facility	64.9 (±16.1)		Aircraft	60.6 (±16.3)
	Shopping Mall	67.4 (±15.1)		Boat	55.9 (±17.7)
	Public Facility	64.5 (±16.0)		Other Public Transport	53.2 (±26.8)
	Other Indoor Spaces	64.1 (±19.0)			

† Mean (± Standard deviation)

Table S15: Principal component analysis of self-report habit index in indoor spaces

	Married People*	Have at least one child**	60–69 years***	≥70 years***	Shopping mall†	Public facility‡
	**Component 1**	**Component 1**	**Component 1**	**Component 1**	**Component 1**	**Component 1**
Eigenvalue	7.9	8.1	8.1	8.1	8.0	7.8
Variance explained (%)	65.75	67.17	67.56	67.88	66.54	64.63
Item loadings						
1. I do frequently.	0.81	0.83	0.81	0.83	0.81	0.85
2. I do automatically.	0.84	0.85	0.85	0.84	0.84	0.8
3. I do without having to consciously remember.	0.87	0.89	0.89	0.89	0.88	0.91
4. that makes me feel weird if I do not do it.	0.81	0.81	0.82	0.84	0.82	0.82
5. I do without thinking.	0.88	0.88	0.87	0.88	0.87	0.75
6. that would require effort not to do it.	0.58	0.57	0.61	0.57	0.59	0.53
7. that belongs to my (daily, weekly, monthly) routine.	0.83	0.85	0.83	0.85	0.85	0.85
8. I start doing before I realize I'm doing it.	0.8	0.79	0.83	0.76	0.79	0.82
9. I would find hard not to do.	0.86	0.87	0.88	0.87	0.85	0.88
10. I have no need to think about doing.	0.79	0.79	0.78	0.79	0.8	0.72
11. that's typically "me."	0.78	0.79	0.82	0.84	0.79	0.78
12. I have been doing for a long time.	0.85	0.87	0.85	0.87	0.85	0.87

* The number of married people is 1,440.

** The number of people who have at least one child is 1,247.

*** The 60–69 and ≥70 years groups have the same sample size of 440.

† The number of people who frequently visit shopping malls is 935.

‡ The number of people who frequently visit public facilities is 91.

Component 1: the first principal component.

Table S16: Principal component analysis of self-report habit index on public transport

	People who are 20–29 years old*				People who frequently use trains**			
	Before Rotation		After Rotation		Before Rotation		After Rotation	
	Component 1	Component 2	Component 1	Component 2	Component 1	Component 2	Component 1	Component 2
Eigenvalue	8.3	1.0	5.5	3.3	8.4	1.0	5.1	3.7
Variance explained (%)	69.45	8.46	45.59	27.46	69.74	7.98	42.20	30.71
Item loadings								
1. I do frequently.	0.84	-0.30	0.83	-	0.82	-0.35	0.78	-
2. I do automatically.	0.88	-0.20	0.82	-	0.88	-0.23	0.85	-
3. I do without having to consciously remember.	0.90	-0.21	0.84	-	0.90	-0.22	0.84	-
4. that makes me feel weird if I do not do it.	0.84	0.27	0.51	0.70	0.84	0.28	0.48	0.73
5. I do without thinking.	0.91	-0.07	0.77	0.50	0.90	-0.08	0.76	0.50
6. that would require effort not to do it.	0.65	0.57	-	0.70	0.67	0.53	-	0.65
7. that belongs to my (daily, weekly, monthly) routine.	0.88	-0.28	0.85	-	0.86	-0.26	0.75	0.41
8. I start doing before I realize I'm doing it.	0.85	-0.06	0.68	0.47	0.82	-0.09	0.66	0.46
9. I would find hard not to do.	0.86	0.19	0.56	0.66	0.87	0.24	0.49	0.76
10. I have no need to think about doing.	0.83	-0.03	0.65	0.48	0.84	0.01	0.61	0.55
11. that's typically "me."	0.67	0.55	-	0.74	0.75	0.47	-	0.78
12. I have been doing for a long time.	0.82	-0.15	0.69	0.41	0.85	-0.13	0.66	0.49

* The 20–29 years group has a sample size of 440.

** The number of people who frequently use trains is 1,709.

Component 1: the first principal component; Component 2: the second principal component; Rotation: maximum likelihood extraction and varimax rotation method. Rotated component loading values < 0.4 are not reported.
